# Father involvement in pregnancy and attachment to their baby: Depression and partner relationships in a sample of Black fathers

**DOI:** 10.1002/imhj.70065

**Published:** 2025-12-29

**Authors:** Alvin Thomas, Tova Walsh, Helenia Quince, Jacob White, Dalvery Blackwell

**Affiliations:** ^1^ School of Human Ecology University of Wisconsin–Madison Madison Wisconsin USA; ^2^ Sandra Rosenbaum School of Social Work University of Wisconsin–Madison Madison Wisconsin USA; ^3^ African American Breastfeeding Network Milwaukee Wisconsin USA

**Keywords:** attachment, Black fathers, father involvement, parent relationship quality, paternal depression, pregnancy

## Abstract

Black fathers, particularly those who are low‐income, unmarried, and from minoritized communities, face significant barriers to full participation in their children's lives. Prenatal challenges include scheduling conflicts, living arrangements, and relationship dynamics with the child's mother. These factors critically impact early paternal involvement and infant bonding. Despite the importance of father involvement, research on Black fathers' prenatal involvement and infant attachment remains limited. This study examines 75 Black fathers in the United States, investigating how relationship quality and paternal depressive symptoms influence prenatal involvement and early infant attachment. The research seeks to illuminate the complex interpersonal dynamics that shape paternal involvement during pregnancy and infancy, and addresses a crucial gap in understanding Black fatherhood experiences. We found that fathers’ depressive symptoms were negatively associated with the quality of their relationship with the mother of their child (B = −1.398, SE = .524, *p* = .009, LLCI = −2.443, ULCI = −.353). The findings suggest interparental relationship quality and paternal depressive symptoms are key interpersonal and personal factors that are associated with father involvement in pregnancy and early infant attachment with the baby.

## 1 FATHER INVOLVEMENT IN PREGNANCY AND ATTACHMENT TO THEIR BABY: DEPRESSION AND PARTNER RELATIONSHIPS IN A SAMPLE OF BLACK FATHERS

Fathers play an important role in child health and development, beginning even before birth. Father involvement during pregnancy is associated with improved maternal and infant health outcomes, as well as benefits to men's own well‐being. (Alio et al., 2010, Alio et al., 2013, Plantin et al., 2011, Salihu et al., 2014) While measures of father involvement have often been limited to marital status or presence/absence at birth, increasingly, researchers are assessing prenatal father involvement in more comprehensive ways, including provision of financial, instrumental, and social support during pregnancy and participation in prenatal care, labor, and delivery. (Lee et al., 2018) These types of involvement have been associated with earlier maternal initiation of prenatal care, reduction in maternal smoking, lower risk of infant low birth weight, and greater likelihood of continued father involvement as the infant grows. (Lee et al., 2018, Martin et al., 2007, Yogman & Eppel, 2022, Shannon et al., 2009, Yogman et al., 2016, Teitler, 2001)

Father‐infant relationships have important developmental implications (Teitler, 2001), and the prenatal period is an important time for relationship formation. (Yogman et al., 2016, Teitler, 2001, Brown & Aytuglu, 2020) Fathers begin to establish an emotional connection to the expected infant during pregnancy, and this relationship, known as paternal prenatal attachment, develops over the course of pregnancy and informs the father‐infant relationship that begins at birth. (Brandon et al., 2009, Condon & Corkindale, 1997, Condon et al., 2013) In general, we know very little about paternal prenatal attachment and paternal‐infant attachment. (Condon et al., 2013, Tolman et al., 2021) While there is scant research on the association between prenatal and postnatal father‐infant attachment, existing research consistently demonstrates that attachment increases over the course of pregnancy and prenatal attachment is associated with postnatal attachment, albeit moderately. (Condon & Corkindale, 1997, Condon et al., 2013, Weaver & Cranley, 1983) Family systems theory frames the family as an interconnected network of systems that influence individual development. (Minuchin, 1985) Each player and subsystem within the family (e.g., parent‐infant relationship) continually adjusts to maintain family functioning. (Cox & Paley, 2003)

**Key findings**

1. Fathers' depressive symptoms are negatively related to the quality of the relationship with the child's mother.
2. Interparental relationship quality has indirect associations to fathers' pregnancy involvement and early infant attachment.
3. Depressive symptoms and relationship quality are crucially associated with paternal involvement.

**Statement of relevance**

This study provides critical insights into the complex psychological dynamics of Black fatherhood, revealing how paternal depressive symptoms and partner relationships are associated with early father‐infant attachment and involvement. By highlighting the intricate interpersonal factors that are related to paternal involvement during pregnancy and infancy, the research offers clinicians and researchers a nuanced understanding of supporting father‐infant relationships in Black families, a demographic historically underrepresented in infant depressive symptoms literature.

In this study, paternal attachment to the infant is an outcome which is influenced by player characteristics and subsystems–paternal depressive symptoms and interparental relationship quality. Paternal prenatal attachment has been positively correlated with interparental relationship quality as perceived by the expectant father during pregnancy (Brandon et al., 2009, Weaver & Cranley, 1983, Condon et al., 2008) and with postpartum paternal‐infant attachment. (Cunen et al., 2017) This is consistent with research that shows the influence of mothers on father‐infant relationships (Schoppe‐Sullivan et al., 2008) and the association between father involvement during pregnancy and greater involvement with his child over time. (Cabrera et al., 2008)

A growing body of research highlights fathers’ vulnerability to depression in the period surrounding the birth of a baby. The literature demonstrates that paternal depression can negatively affect parenting, family relationships, and child development. (Walsh et al., 2020) A meta‐analysis of paternal perinatal depression reported prevalence rates between 2%–25%. (Giallo et al., 2014) Most studies examining the association between prenatal depression and prenatal attachment have found that prenatal depressive symptoms are negatively associated with prenatal attachment. However, few studies have included fathers, and findings related to fathers have been mixed. (Hjelmstedt et al., 2007, Mercer et al., 1988, Rollè et al., 2020, Vreeswijk et al., 2014) Even fewer studies have examined these issues among Black fathers.

There is an overall scarcity of research on men's experiences, their contributions in the prenatal period, and the neglect of men in research on early parent‐child relationship formation. (Tolman et al., 2021) Compounding this, we know even less about prenatal involvement and early bonding among Black fathers and their children because the limited research in this area has largely included White fathers. The role of fathers in pregnancy and as a support to mothers among lower‐income, married and unmarried, racially and ethnically diverse parents is thus not well understood. (Garfield & Isacco, 2009)

Despite persistent, damaging myths to the contrary, evidence demonstrates that Black fathers are more involved than White and other fathers in the United States (U.S.). (Jones & Mosher, 2013) Among Black expectant parents, relationship quality characterized by closer relationships between mother and father is associated with lower maternal stress. (Eboh et al., 2018) Additionally, higher levels of conflict are associated with a higher risk of depression for both parents (Caldwell et al., 2018) and a higher risk of pre‐term birth. (Nutor et al., 2018) Thus, interparental relationship quality can have strong impacts on fathers and paternal involvement in general (Brown & Aytuglu, 2020, Ferketich & Mercer, 1995, Chen et al., 2019). Relationship quality–the negative or positive valence of interactions between the parents of the infant is critically associated with paternal attachment to their infant, and the family system as a whole.

Using the tenets of family systems theory, this study examines the association between fathers’ involvement in pregnancy–the level to which fathers engage in daily activities that support the mother throughout the pregnancy period–and fathers’ attachment with their baby. The study also explores the influence of fathers’ level of depressive symptoms and interparental relationship quality on the link between involvement in pregnancy and attachment to the infant. The study limited exploration of relationship quality to being between co‐parents who are and are not romantic partners but are biological parents of the same infant. The study was guided by two hypotheses: (1) there will be indirect associations among relationship quality, fathers’ depressive symptoms, and involvement in pregnancy; (2) there will also be indirect associations among relationship quality, fathers’ involvement in the pregnancy, and attachment to the infant.

## 2 METHODS

The data for these analyses come from a study conducted in 2021 in partnership with the (removed), a community organization dedicated to championing maternal infant health and breastfeeding equity. We conducted online surveys of 75 Black mothers and 75 Black fathers. To be included in the study, participants need to meet the following criteria: (1) self‐identified as Black or African‐American, (2) living in Milwaukee, and (3) expecting a baby and/or parent to an infant. Milwaukee is one of the most racially segregated cities in the U.S. (Loyd & Bonds, 2018) The African American Breastfeeding Network led participant recruitment, disseminating study information to clients (e.g., families receiving doula services or breastfeeding support) and to the community more broadly at community events. The study information included a link to the survey, which was administered on the online platform Qualtrics. Potential participants first completed an eligibility screening. Eligible participants were then provided with informed consent information and advanced to the survey. The objective of the larger study was to identify new, father‐inclusive strategies to improve outcomes for Black mothers, babies, fathers, and families. In this paper, we use survey data collected from fathers (*N* = 75) to examine interparental relationship quality, paternal depressive symptoms, fathers’ involvement in pregnancy, and paternal attachment to their baby. For sample characteristics, see Table 1. Fathers completed the survey online. The survey took 20–30 min to complete, and participants were compensated $25 in the form of an electronic gift card. The University of Wisconsin–Madison's Institutional Review Board designated the study as exempt research. No significant differences were found between expectant and recent fathers, so the sample of 75 fathers was combined for these analyses as with previous manuscripts from these data. (Walsh et al., 2023)

**TABLE 1:** Sociodemographic characteristics of the sample.

Variables	*n*	*%*
**Age in years**		
18–25	11	14.7
26–34	48	64
35–43	13	17.3
≥44	3	4
**Education status**		
Some high school	6	8
High school diploma/GED	13	17.3
Some college/trade school	40	53.4
Bachelor's degree	12	16
Advanced degree (e.g., PhD, MD, JD)	4	5.3
**Work status**		
Unemployed	6	8
Part‐time employment	12	16
Full‐time employment	57	76
**Relationship status**		
Single	22	29.3
Married	29	38.7
Separated	3	4
Living with partner	21	28
**Expectant/new parent**		
Expecting a baby	54	72
Parent to an infant	21	28
**Father's household income**		
≤ $24,999	6	8
$25,000–$49,999	39	52
$50,000–$74,999	21	28
$75,000–$99,999	5	6.7
≥$100,000	4	5.3

### 2.1 Measures

#### 2.1.1 Relationship Quality

We measured fathers’ perception of their relationship with their baby's mother using three items. Items included “How close or distant is your relationship with the mother of your baby?”, “In general, how often do you think that things between you and the mother of your baby are going well?”, and “How often do you feel like part of a team with the mother of your baby?” The first item was measured on a 6‐point Likert scale from 1 = extremely distant to 6 = extremely close. The Likert scale for the second and third questions was from 1 = never to 6 = all the time. These items were crafted together with the African American Breastfeeding Network, which advised on cultural relevance and accessibility of the items. We were intentional about designing items to be relevant to assessing the quality of the relationship between co‐parents who are and are not romantic partners and are biological parents for the infant. We calculated a total score by combining the 3 items for a total possible score of 18, where higher scores indicated a stronger relationship between fathers and mothers. The scale had a Cronbach alpha of .848 in this sample.

#### 2.1.2 Depressive Symptoms

We assessed fathers’ depressive symptoms using the Patient Health Questionnaire‐2 (PHQ‐2). (Kroenke et al., 2003) Participants were asked. “Over the last 2 weeks, how often have you been bothered by any of the following problems?” and responded to the following two items: “Little interest or pleasure in doing things” and “Feeling down, depressed or hopeless”. Both items were measured on a 4‐point Likert scale where 0 = not at all and 3 = nearly every day. We calculated a total score from 0 to 6, with higher scores representing more depressive symptoms. Cut‐off scores of ≥3 is indicative of depression on the PHQ‐2. The two items had a Pearson correlation of *r* = .404, *p* < .001. The PHQ‐2 has demonstrated excellent discrimiative validity and an optimal level of clinical sensitivity. Additionally, psychometric tests of this measure had demonstrated good test‐retest reliability and strong internal consistency. (Staples et al., 2019) To the best of our knowledge this is the only study that features the use of the PHQ‐2 among Black or African American fathers.

#### 2.1.3 Involvement in pregnancy

We measured fathers’ involvement in pregnancy using 8 items measured on a Likert scale from 1 = never to 5 = all of the time. The items were the same for expectant fathers and fathers of infants, only modified to reflect what the dad is currently doing or did previously during pregnancy. Items included “I help with the household tasks that were done by my partner before pregnancy” and “I support my partner's general health and well‐being (e.g., prepare healthy food)”. These items were developed together with [removed], who advised on cultural relevance and accessibility of the items. Instructions preceding these items clarified that here the word “partner” refers to the father's partner in pregnancy, who may or may not be the father's romantic partner. We summed the items for a total possible score of 40, with higher scores meaning greater father involvement in the pregnancy. The scale had a Cronbach alpha of .785 in this sample.

#### 2.1.4 Attachment to the infant

We assessed fathers’ attachment to their infant/developing child using 4 items drawn from the Paternal Postnatal Attachment Scale. (Condon et al., 2008) Fathers with babies responded to items like, “Over the last two weeks I would describe my feelings for the baby as:”, where response options included dislike, no strong feelings toward the baby, slight affection, moderate affection, and intense affection. Fathers who were expecting babies responded to questions like, “Over the past two weeks my feelings about the developing baby have been:”, where response options included very positive, mainly positive, mixed positive and negative, mainly negative, very negative. All items were scored on a Likert scale from 1 to 5, with higher scores representing greater paternal attachment. We summed scores for the 4 items for a total possible score of 20, with higher scores meaning greater paternal attachment. The scale had a Cronbach alpha of .844 in this sample. PAAS scales have satisfactory reliability–Cronbach's alpha >0.8(Condon, 1993).

#### 2.1.5 Covariates

Fathers’ age was reported as a continuous variable. Household income was measured on a Likert scale where 1 = $24,999 or less; 2 = $25,000–$49,999; 3 = $50,000–74,999; 4 = $75,000–99,999; and 5 = $100,000 or more. Time lived with the baby's mother was reported as a continuous variable.

## 3 STATISTICAL ANALYSES

We tested our hypotheses using hierarchical regression models, controlling for sociodemographic characteristics (i.e., father's age, income, and time lived with the baby's mother). We used *SPSS 28* to conduct the analyses. First, we estimated a mediational model testing indirect associations among relationship quality (the mediator) and fathers’ depressive symptoms and involvement in pregnancy for the first hypothesis. Second, we estimated a mediational model with relationship quality as the mediator, to test our second hypothesis that there would be indirect associations among relationship quality, fathers’ involvement in the pregnancy and attachment to their infant. For both analyses, we used the Sobel test to assess the significance of the mediation effect. (Sobel, 1982) For all regression analyses, we used an alpha level of .05 to determine statistical significance. We calculated standardized regression coefficients (β) to assess effect sizes, with values of .10, .30, and .50 representing small, medium, and large effects, respectively. The significance of mediation effects was evaluated using the Sobel test, which tests the null hypothesis that the indirect effect (a × b) equals zero.

## 4 RESULTS

### 4.1 Descriptives

About 79% of fathers were aged 18 to 34 years old, with about 79% having completed some college or technical/trade school or less. About 39% were married to the mother of their child. Over 58% of fathers reported depressive symptoms in the severe range according to PHQ‐2 cutoffs, and 72% were expecting a baby. Concerning the father's involvement in the pregnancy

Relationship quality was positively correlated with involvement in the pregnancy (*r* = .282, *p* = .014). Paternal attachment was also positively correlated with involvement in the pregnancy (*r* = .323, *p* = .005). However, fathers’ depressive symptoms were not significantly correlated with involvement in the pregnancy (*r* = −.217, *p* = .0.61) in this sample of Black fathers. Regarding the father's attachment to the infant, only the father's involvement in the pregnancy was significantly and positively linked to attachment to the infant. Depressive symptoms (*r* = −.097, *p* = .406) and relationship quality (*r* = .180, *p* = .122) were not significantly correlated with attachment to the infant in this sample of Black fathers. The interparental relationship quality was negatively related to the father's depressive symptoms (*r* = −.332, *p* = .004). Table 2 presents the correlations among the variables.

**TABLE 2:** Descriptives, means, and correlations for study variables.

	1	2	3	4	5	6	7
1. Paternal attachment	1						
2. Involvement in pregnancy	.323^**^						
3. Depressive symptoms	−.097	−.217					
4. Relationship quality	.180	.282^*^	−.322^**^				
5. Lived with baby's mother	.103	.093	−.086	.353^**^			
6. Income	.056	.016	−.203	−.021	−.012		
7. Age	.099	−.093	−.248^*^	.086	.114	.190	1
Mean	15.77	27.13	.733	14.40	3.66	‐	‐
S.D.	5.47	7.35	.72	3.33	.60	‐	‐

* *p*<.05;

** *p*<.001

#### 4.1.1 Multivariate Analyses

Our first hypothesis, that relationship quality will mediate the link between fathers’ depressive symptoms and involvement in pregnancy, was not confirmed. See Table 3a. To rule out a bidirectional effect, we also tested the reverse of this hypothesis, and this too was not significant. The indirect association of depressive symptoms with fathers’ involvement in the pregnancy through relationship quality was non‐significant (*B* = −.653, LLCI = −1.636, ULCI = .098). However, the test of the first stage in the mediation hypothesis revealed that fathers’ depressive symptoms were negatively related to the quality of their relationship with the mother of their baby (*B* = −1.398, SE = .524, *p* = .009, LLCI = −2.443, ULCI = −.353). Fathers who reported more depressive symptoms also reported a lower quality of relationship with the mother of their baby. With relationship quality added to the model, neither relationship quality (*B* = .467, SE = .291, *p* = .113, LLCI = −.114, ULCI = 1.048) nor depressive symptoms (*B* = −1.171, SE = 1.312, *p* = .375, LLCI = −3.790, ULCI = 1.448) were significantly associated with fathers’ involvement in the pregnancy.

**TABLE 3a:** Mediation effect of relationship quality on the link between fathers’ depressive symptoms and their involvement in the pregnancy *(N=75)*

		Interparental Relationship Quality		95% CI			Fathers’ involvement in pregnancy		95% CI	
	B	SE	*p*	LL	UL	B	SE	*p*	LL	UL
Depressive Symptoms	−1.398	0.524	0.009	−2.443	−0.353	−1.171	1.312	0.375	−3.790	1.448
Interparental Relationship Quality	−	−	−	−	−	0.467	0.291	0.113	−0.114	1.048
Lived with child	1.761	0.600	0.005	0.563	2.959	−0.098	1.518	0.949	−3.790	2.934
Fathers’ income	−0.277	0.395	0.486	−1.066	0.512	−0.061	0.945	0.949	−1.947	1.826
Baby's age	0.016	0.621	0.980	−1.224	1.256	0.674	1.480	0.650	−2.280	3.629
*R^2^*	0.208	9.316	0.003			0.082	52.863	0.332		
*F*	4.404					1.173				

**TABLE 3b:** Mediation effect of relationship quality on the link between fathers’ involvement in the pregnancy and attachment to their infant *(N=75)*

		Interparental Relationship Quality		95% CI			Father's attachment to their infant		95% CI	
	B	SE	*p*	LL	UL	B	SE	*p*	LL	UL
Fathers’ involvement in pregnancy	0.114	0.049	0.022	0.017	0.211	0.061	0.043	0.156	−0.024	0.147
Interparental Relationship Quality	−	−	−	−	−	0.432	0.102	< 0.000	0.229	0.636
Lived with child	1.914	0.597	0.002	0.724	3.104	0.544	0.543	0.320	−0.539	1.627
Fathers’ income	0.085	0.396	0.830	−0.705	0.875	0.296	0.336	0.382	−0.375	0.967
Baby's age	1.490	0.976	0.132	−0.458	3.437	1.340	0.842	0.116	−0.341	3.021
*R^2^*	0.212	9.275	0.002			0.359	6.680	< 0.000		
*F*	4.641					7.619				

The second hypothesis was confirmed, demonstrating that relationship quality mediated the link between fathers’ involvement in the pregnancy and attachment to their infant. See Table 3b. To rule out a bidirectional effect, we also tested the reverse of this hypothesis, and this was not significant. In keeping with our original hypothesis, however, the indirect link between involvement in the pregnancy and fathers’ attachment to their infant through relationship quality was positive and significant (*B* = .049, LLCI = .009, ULCI = .127). The test of this hypothesis revealed that fathers’ involvement in the pregnancy (*B* = .114, SE = .049, *p* = .022, LLCI = .017, ULCI = .211), and living with the mother of their infant (*B* = 1.914, SE = .597, *p* = .002, LLCI = .724, ULCI = 3.104) were both positively linked to the quality of their relationship with the mother of their baby. Results of the Sobel test also revealed a significant mediation effect (z 2.039, *p* = 0.041). Fathers who reported greater involvement in the pregnancy and sharing a home with the mother of their infant also reported a better relationship with the mother of their infant. With relationship quality as part of the model, fathers’ involvement in the pregnancy (*B* = .061, SE = .043, *p* = .156, LLCI = −.024, ULCI = .147) became non‐significant, but relationship quality was significantly associated (*B* = .432, SE = .102, *p* < .001, LLCI = .229, ULCI = .636) with fathers’ attachment to the baby.

## 5 DISCUSSION

This study was predicated on addressing critical gaps in the father‐infant attachment literature by exploring Black fathers’ involvement in pregnancy and attachment to their infant.

We focused on the relationships among paternal depression, interparental relationship quality, and Black fathers’ involvement in pregnancy as key subsystems of the family system to better understand fathers’ attachment to their infant. We found that among the fathers in this study, relationship quality is key to fathers’ involvement in pregnancy and their attachment to their children. Additionally, there were indirect associations among relationship quality, fathers’ involvement in the pregnancy, and attachment to their child.

Though rates of paternal depression are elevated during the perinatal period (Walsh et al., 2020, Hjelmstedt et al., 2007) and the effects of depression on couple and parent‐infant relationships are widely demonstrated (Mercer et al., 1988), relatively little is known about how these dynamics might look for Black fathers. Our results suggest that relationship quality did not demonstrate indirect associations with the link between fathers’ depressive symptoms and involvement in pregnancy among the fathers in this study; however, the results confirm a negative effect of paternal depression on relationship quality. Compared to depressed mothers, depressed fathers are more likely to “mask” their symptoms through avoidant or numbing behaviors and show increased anger or irritability rather than sadness. (Walsh et al., 2020, Fisher, 2017) These symptoms may compromise the father's ability to provide emotional support and actively engage in parenting tasks, causing strain in the couple's relationship. The strain may be greater if fathers’ depression is not recognized and understood as such. Paternal depression may also be associated with fathers' pregnancy involvement through individual psychological mechanisms such as reduced self‐efficacy and negative thinking patterns, rather than operating through relationship quality. This suggests that during pregnancy, individual factors may have a more direct impact on father engagement than relational dynamics between partners. Still, the contribution of paternal depression to a decline in relationship quality highlights the importance of addressing paternal mental health for the well‐being of the entire family unit.

Paternal attachment is closely linked to the interparental relationship quality during pregnancy and postpartum (Condon & Corkindale, 1997, Condon et al., 2013, Tolman et al., 2021), but less is known about this association for Black fathers. Our results suggest that for the Black fathers in this study, the quality of the relationship with the mother of their infant was associated with fathers’ involvement in pregnancy and attachment to their infant. A positive relationship quality between parents was linked to greater involvement of fathers during the pregnancy, including attending prenatal appointments, offering emotional support, and participating in decision‐making processes. This active involvement may enhance the father's sense of connection and investment in the pregnancy. Qualitative research has found that father involvement in pregnancy is highly meaningful and important to both mothers and fathers (Tolman et al., 2021), and Black emphasizes the importance of being a team in pregnancy and parenting regardless of their relationship status. (Walsh et al., 2023) It is important to note that two‐thirds of the sample were married or cohabiting with the mother of their baby. Our findings highlight relationship quality as a critical resource that providers can engage in pre‐ and perinatal care settings. Therefore, nurturing a healthy and supportive relationship between parents during pregnancy not only benefits the parents but also serves as a vital factor in facilitating fathers' attachment to their infant, ultimately contributing to positive parenting outcomes and family well‐being.

### 5.1 Limitations

There are a few limitations to the current study. The survey sample (*N* = 75) was relatively small and restricted to Black men from (removed). This limits the statistical power and generalizability of findings, while the focus on fathers from a very specific location in the United States restricts its generalizability to Black fathers from other localities. However, this study's contribution to understanding how depression and relationship quality are related to Black fathers’ attachment to their children offers a foundation for continued exploration of these relationships among Black fathers in other localities. The study does not include a measure of pregnancy intention, and there is significant literature on how that affects parental motivations and could affect mother‐father‐child relationships. There is a need for future studies to explore father involvement, attachment, and interparental relationships in the context of pregnancy intention. Similarly, future research is needed to build on the present analysis by incorporating attention to instability and adversity. Additionally, testing indirect associations with a cross‐sectional design, while not novel, is an acceptable limitation, especially for studies that are establishing conceptual relationships in largely understudied populations. Thus, the study highlights the critical need for longitudinal studies on this topic especially since mental health and relationships can fluctuate over time. Still, the current study offers important preliminary insight, and future research is needed to extend knowledge in this area.

The study uses data gathered directly from fathers, thus featuring the voice of Black fathers, however, future studies could incorporate data from multiple informants, such as observations of father‐infant interaction and maternal report of relationship quality, in addition to father report. The study design precludes calculating a response rate; we did not recruit participants individually, rather the African American Breastfeeding Network disseminated study information widely to their clients and across the community, and interested individuals reached out to the study team. Additionally, while fathers experiences at the prenatal and postpartum periods generally differ, in this sample of Black fathers we did not observe any significant differences on the key variables. This may be a reflection of the sample size limitation, and suggest the need for exploring these relationships in larger samples to see how this may differ at different points in the pregnancy experience. Still, our findings contribute to a better understanding of factors that impact early infant attachment with children among Black fathers. We highlight the imperative of additional research to further elucidate the involvement, experiences, and influence of Black fathers in the perinatal period.

## 6 CONCLUSIONS AND IMPLICATIONS

Black fathers’ involvement in pregnancy and their attachment to their babies is influenced by multiple interconnected factors. The role of relationship quality in the indirect relationships between fathers' depressive symptoms and involvement in pregnancy, as well as the link between involvement in pregnancy and attachment to their infant, highlights the importance of nurturing supportive and positive relationships between parents during this crucial period. For families, understanding the critical role of relationship quality and depressive symptoms can guide them in building a supportive and nurturing environment. Open communication, empathy, and seeking professional help when needed can enhance relationship quality and mitigate the negative effects of depressive symptoms. Fathers can actively participate in the pregnancy journey, fostering a sense of connection and attachment to their infant. These findings also underscore the need for targeted interventions and resources specifically designed to support Black fathers' depressive symptoms and involvement during pregnancy.

Mental health professionals, including licensed clinical social workers, clinical psychologists, and psychiatrists trained in perinatal mental health, can provide individual and couples and family therapy to address depressive symptoms and improve relationship dynamics. Obstetricians, midwives, and prenatal care providers can integrate father‐inclusive practices into routine prenatal visits, encouraging active participation and addressing interparental relationship concerns. At the grassroots level, community health workers and peer support specialists with lived experience can offer culturally relevant guidance and connect fathers to appropriate resources. Doulas and childbirth educators can facilitate father involvement through specialized classes and support groups designed for engaging expectant Black fathers.

Public health departments, community‐based organizations, and faith‐based institutions can implement and coordinate culturally sensitive programs that address the unique experiences and challenges faced by Black fathers. Social workers specializing in family services can provide case management and connect families to comprehensive support networks. These interventions can be instrumental in promoting positive relationship dynamics, enhancing involvement in pregnancy, and facilitating secure attachments. Supporting Black fathers' depressive symptoms and involvement during pregnancy not only benefits fathers themselves but also contributes to the well‐being and development of their children, promoting stronger, more resilient families.

## CONFLICT OF INTEREST STATEMENT

The authors declare no competing interests or conflicts of interest.

## FUNDING INFORMATION

Funding for this project was provided by the UW School of Medicine and Public Health from the Wisconsin Partnership Program through a grant to the UW Institute for Clinical and Translational Research. Research reported in this publication was supported in part by the Eunice Kennedy Shriver National Institute of Child Health & Human Development, the Office of Research on Women's Health, Building Interdisciplinary Research Careers in Women's Health (BIRCWH) program, the Office of The Director, National Institutes of Health and the National Cancer Institute, under Award Number K12HD055894.

## Data Availability

The datasets generated during this study are not publicly available due to participant privacy considerations but are available from the corresponding author upon reasonable request.
